# Supplementary material for: OpenMM 7: Rapid development of high performance algorithms for molecular dynamics
Source: PLoS Comput Biol. 2017 Jul 26;13(7):e1005659. doi: 10.1371/journal.pcbi.1005659 (PMC5549999; doi:10.1371/journal.pcbi.1005659)
Supplement: S1 Source Code — (ZIP) [file pcbi.1005659.s002.zip › OpenMM-7.0.1-Source/docs/api-c++/generated/OpenMM.CustomCentroidBondForce.html]

CustomCentroidBondForce — OpenMM @OPENMM\_MAJOR\_VERSION@.@OPENMM\_MINOR\_VERSION@ documentation


# CustomCentroidBondForce¶

*class* `OpenMM::``CustomCentroidBondForce`¶
:   This class is similar to `CustomCompoundBondForce`, but instead of applying forces between individual particles, it applies them between the centers of groups of particles. This is useful for a variety of purposes, such as restraints to keep two molecules from moving too far apart.

    When using this class, you define groups of particles, and the center of each group is calculated as a weighted average of the particle positions. By default, the particle masses are used as weights, so the center position is the center of mass. You can optionally specify different weights to use. You then add bonds just as with `CustomCompoundBondForce`, but instead of specifying the particles that make up a bond, you specify the groups.

    When creating a `CustomCentroidBondForce`, you specify the number of groups involved in a bond, and an expression for the energy of each bond. It may depend on the center positions of individual groups, the distances between the centers of pairs of groups, the angles formed by sets of three groups, and the dihedral angles formed by sets of four groups.

    We refer to the groups in a bond as g1, g2, g3, etc. For each bond, `CustomCentroidBondForce` evaluates a user supplied algebraic expression to determine the interaction energy. The expression may depend on the following variables and functions:

    > - x1, y1, z1, x2, y2, z2, etc.: The x, y, and z coordinates of the centers of the groups. For example, x1 is the x coordinate of the center of group g1, and y3 is the y coordinate of the center of group g3.
    > - distance(g1, g2): the distance between the centers of groups g1 and g2 (where “g1” and “g2” may be replaced by the names of whichever groups you want to calculate the distance between).
    > - angle(g1, g2, g3): the angle formed by the centers of the three specified groups.
    > - dihedral(g1, g2, g3, g4): the dihedral angle formed by the centers of the four specified groups.

    The expression also may involve tabulated functions, and may depend on arbitrary global and per-bond parameters.

    To use this class, create a `CustomCentroidBondForce` object, passing an algebraic expression to the constructor that defines the interaction energy of each bond. Then call `addPerBondParameter()` to define per-bond parameters and `addGlobalParameter()` to define global parameters. The values of per-bond parameters are specified as part of the system definition, while values of global parameters may be modified during a simulation by calling `Context::setParameter()`.

    Next call `addGroup()` to define the particle groups. Each group is specified by the particles it contains, and the weights to use when computing the center position.

    Then call `addBond()` to define bonds and specify their parameter values. After a bond has been added, you can modify its parameters by calling `setBondParameters()`. This will have no effect on Contexts that already exist unless you call `updateParametersInContext()`.

    As an example, the following code creates a `CustomCentroidBondForce` that implements a harmonic force between the centers of mass of two groups of particles.

    ```
    CustomCentroidBondForce* force = new CustomCentroidBondForce(2, "0.5*k*distance(g1,g2)^2");
    force->addPerBondParameter("k");
    force->addGroup(particles1);
    force->addGroup(particles2);
    vector<int> bondGroups;
    bondGroups.push_back(0);
    bondGroups.push_back(1);
    vector<double> bondParameters;
    bondParameters.push_back(k);
    force->addBond(bondGroups, bondParameters);
    ```

    Expressions may involve the operators + (add), - (subtract), \* (multiply), / (divide), and ^ (power), and the following functions: sqrt, exp, log, sin, cos, sec, csc, tan, cot, asin, acos, atan, sinh, cosh, tanh, erf, erfc, min, max, abs, floor, ceil, step, delta, select. All trigonometric functions are defined in radians, and log is the natural logarithm. step(x) = 0 if x is less than 0, 1 otherwise. delta(x) = 1 if x is 0, 0 otherwise. select(x,y,z) = z if x = 0, y otherwise.

    In addition, you can call `addTabulatedFunction()` to define a new function based on tabulated values. You specify the function by creating a `TabulatedFunction` object. That function can then appear in the expression.

    Methods

    |  |  |
    | --- | --- |
    | `CustomCentroidBondForce` | Create a `CustomCentroidBondForce`. |
    | `~CustomCentroidBondForce` |  |
    | `getNumGroupsPerBond` | Get the number of groups used to define each bond. |
    | `getNumGroups` | Get the number of particle groups that have been defined. |
    | `getNumBonds` | Get the number of bonds for which force field parameters have been defined. |
    | `getNumPerBondParameters` | Get the number of per-bond parameters that the interaction depends on. |
    | `getNumGlobalParameters` | Get the number of global parameters that the interaction depends on. |
    | `getNumTabulatedFunctions` | Get the number of tabulated functions that have been defined. |
    | `getNumFunctions` | Get the number of tabulated functions that have been defined. |
    | `getEnergyFunction` | Get the algebraic expression that gives the interaction energy of each bond |
    | `setEnergyFunction` | Set the algebraic expression that gives the interaction energy of each bond |
    | `addPerBondParameter` | Add a new per-bond parameter that the interaction may depend on. |
    | `getPerBondParameterName` | Get the name of a per-bond parameter. |
    | `setPerBondParameterName` | Set the name of a per-bond parameter. |
    | `addGlobalParameter` | Add a new global parameter that the interaction may depend on. |
    | `getGlobalParameterName` | Get the name of a global parameter. |
    | `setGlobalParameterName` | Set the name of a global parameter. |
    | `getGlobalParameterDefaultValue` | Get the default value of a global parameter. |
    | `setGlobalParameterDefaultValue` | Set the default value of a global parameter. |
    | `addGroup` | Add a particle group. |
    | `getGroupParameters` | Get the properties of a group. |
    | `setGroupParameters` | Set the properties of a group. |
    | `addBond` | Add a bond to the force |
    | `getBondParameters` | Get the properties of a bond. |
    | `setBondParameters` | Set the properties of a bond. |
    | `addTabulatedFunction` | Add a tabulated function that may appear in the energy expression. |
    | `getTabulatedFunction` | Get a const reference to a tabulated function that may appear in the energy expression. |
    | `getTabulatedFunction` | Get a reference to a tabulated function that may appear in the energy expression. |
    | `getTabulatedFunctionName` | Get the name of a tabulated function that may appear in the energy expression. |
    | `updateParametersInContext` | Update the per-bond parameters in a `Context` to match those stored in this `Force` object. |
    | `usesPeriodicBoundaryConditions` | Returns whether or not this force makes use of periodic boundary conditions. |

    `CustomCentroidBondForce`(int *numGroups*, *const* std::string &*energy*)¶
    :   Create a `CustomCentroidBondForce`.

        |  |  |
        | --- | --- |
        | Parameters: |  |

        - **numGroups** – the number of groups used to define each bond
        - **energy** – an algebraic expression giving the interaction energy of each bond as a function of particle positions, inter-particle distances, angles, and dihedrals, and any global and per-bond parameters

    `~CustomCentroidBondForce`()¶

    int `getNumGroupsPerBond`() *const*¶
    :   Get the number of groups used to define each bond.

    int `getNumGroups`() *const*¶
    :   Get the number of particle groups that have been defined.

    int `getNumBonds`() *const*¶
    :   Get the number of bonds for which force field parameters have been defined.

    int `getNumPerBondParameters`() *const*¶
    :   Get the number of per-bond parameters that the interaction depends on.

    int `getNumGlobalParameters`() *const*¶
    :   Get the number of global parameters that the interaction depends on.

    int `getNumTabulatedFunctions`() *const*¶
    :   Get the number of tabulated functions that have been defined.

    int `getNumFunctions`() *const*¶
    :   Get the number of tabulated functions that have been defined.

        Deprecated

        This method exists only for backward compatibility. Use `getNumTabulatedFunctions()` instead.

    *const* std::string &`getEnergyFunction`() *const*¶
    :   Get the algebraic expression that gives the interaction energy of each bond

    void `setEnergyFunction`(*const* std::string &*energy*)¶
    :   Set the algebraic expression that gives the interaction energy of each bond

    int `addPerBondParameter`(*const* std::string &*name*)¶
    :   Add a new per-bond parameter that the interaction may depend on.

        |  |  |
        | --- | --- |
        | Parameters: |  |

        - **name** – the name of the parameter

        |  |  |
        | --- | --- |
        | Returns: | the index of the parameter that was added |

    *const* std::string &`getPerBondParameterName`(int *index*) *const*¶
    :   Get the name of a per-bond parameter.

        |  |  |
        | --- | --- |
        | Parameters: |  |

        - **index** – the index of the parameter for which to get the name

        |  |  |
        | --- | --- |
        | Returns: | the parameter name |

    void `setPerBondParameterName`(int *index*, *const* std::string &*name*)¶
    :   Set the name of a per-bond parameter.

        |  |  |
        | --- | --- |
        | Parameters: |  |

        - **index** – the index of the parameter for which to set the name
        - **name** – the name of the parameter

    int `addGlobalParameter`(*const* std::string &*name*, double *defaultValue*)¶
    :   Add a new global parameter that the interaction may depend on.

        |  |  |
        | --- | --- |
        | Parameters: |  |

        - **name** – the name of the parameter
        - **defaultValue** – the default value of the parameter

        |  |  |
        | --- | --- |
        | Returns: | the index of the parameter that was added |

    *const* std::string &`getGlobalParameterName`(int *index*) *const*¶
    :   Get the name of a global parameter.

        |  |  |
        | --- | --- |
        | Parameters: |  |

        - **index** – the index of the parameter for which to get the name

        |  |  |
        | --- | --- |
        | Returns: | the parameter name |

    void `setGlobalParameterName`(int *index*, *const* std::string &*name*)¶
    :   Set the name of a global parameter.

        |  |  |
        | --- | --- |
        | Parameters: |  |

        - **index** – the index of the parameter for which to set the name
        - **name** – the name of the parameter

    double `getGlobalParameterDefaultValue`(int *index*) *const*¶
    :   Get the default value of a global parameter.

        |  |  |
        | --- | --- |
        | Parameters: |  |

        - **index** – the index of the parameter for which to get the default value

        |  |  |
        | --- | --- |
        | Returns: | the parameter default value |

    void `setGlobalParameterDefaultValue`(int *index*, double *defaultValue*)¶
    :   Set the default value of a global parameter.

        |  |  |
        | --- | --- |
        | Parameters: |  |

        - **index** – the index of the parameter for which to set the default value
        - **defaultValue** – the default value of the parameter

    int `addGroup`(*const* std::vector<int> &*particles*, *const* std::vector<double> &*weights* = std::vector< double >())¶
    :   Add a particle group.

        |  |  |
        | --- | --- |
        | Parameters: |  |

        - **particles** – the indices of the particles to include in the group
        - **weights** – the weight to use for each particle when computing the center position. If this is omitted, then particle masses will be used as weights.

        |  |  |
        | --- | --- |
        | Returns: | the index of the group that was added |

    void `getGroupParameters`(int *index*, std::vector<int> &*particles*, std::vector<double> &*weights*) *const*¶
    :   Get the properties of a group.

        |  |  |
        | --- | --- |
        | Parameters: |  |

        - **index** – the index of the group to get
        - **particles** – [out] the indices of the particles in the group
        - **weights** – [out] the weight used for each particle when computing the center position. If no weights were specified, this vector will be empty indicating that particle masses should be used as weights.

    void `setGroupParameters`(int *index*, *const* std::vector<int> &*particles*, *const* std::vector<double> &*weights* = std::vector< double >())¶
    :   Set the properties of a group.

        |  |  |
        | --- | --- |
        | Parameters: |  |

        - **index** – the index of the group to set
        - **particles** – the indices of the particles in the group
        - **weights** – the weight to use for each particle when computing the center position. If this is omitted, then particle masses will be used as weights.

    int `addBond`(*const* std::vector<int> &*groups*, *const* std::vector<double> &*parameters* = std::vector< double >())¶
    :   Add a bond to the force

        |  |  |
        | --- | --- |
        | Parameters: |  |

        - **groups** – the indices of the groups the bond depends on
        - **parameters** – the list of per-bond parameter values for the new bond

        |  |  |
        | --- | --- |
        | Returns: | the index of the bond that was added |

    void `getBondParameters`(int *index*, std::vector<int> &*groups*, std::vector<double> &*parameters*) *const*¶
    :   Get the properties of a bond.

        |  |  |
        | --- | --- |
        | Parameters: |  |

        - **index** – the index of the bond to get
        - **groups** – [out] the indices of the groups in the bond
        - **parameters** – [out] the list of per-bond parameter values for the bond

    void `setBondParameters`(int *index*, *const* std::vector<int> &*groups*, *const* std::vector<double> &*parameters* = std::vector< double >())¶
    :   Set the properties of a bond.

        |  |  |
        | --- | --- |
        | Parameters: |  |

        - **index** – the index of the bond to set
        - **groups** – the indices of the groups in the bond
        - **parameters** – the list of per-bond parameter values for the bond

    int `addTabulatedFunction`(*const* std::string &*name*, TabulatedFunction \**function*)¶
    :   Add a tabulated function that may appear in the energy expression.

        |  |  |
        | --- | --- |
        | Parameters: |  |

        - **name** – the name of the function as it appears in expressions
        - **function** – a `TabulatedFunction` object defining the function. The `TabulatedFunction` should have been created on the heap with the “new” operator. The `Force` takes over ownership of it, and deletes it when the `Force` itself is deleted.

        |  |  |
        | --- | --- |
        | Returns: | the index of the function that was added |

    *const* TabulatedFunction &`getTabulatedFunction`(int *index*) *const*¶
    :   Get a const reference to a tabulated function that may appear in the energy expression.

        |  |  |
        | --- | --- |
        | Parameters: |  |

        - **index** – the index of the function to get

        |  |  |
        | --- | --- |
        | Returns: | the `TabulatedFunction` object defining the function |

    TabulatedFunction &`getTabulatedFunction`(int *index*)¶
    :   Get a reference to a tabulated function that may appear in the energy expression.

        |  |  |
        | --- | --- |
        | Parameters: |  |

        - **index** – the index of the function to get

        |  |  |
        | --- | --- |
        | Returns: | the `TabulatedFunction` object defining the function |

    *const* std::string &`getTabulatedFunctionName`(int *index*) *const*¶
    :   Get the name of a tabulated function that may appear in the energy expression.

        |  |  |
        | --- | --- |
        | Parameters: |  |

        - **index** – the index of the function to get

        |  |  |
        | --- | --- |
        | Returns: | the name of the function as it appears in expressions |

    void `updateParametersInContext`(Context &*context*)¶
    :   Update the per-bond parameters in a `Context` to match those stored in this `Force` object. This method provides an efficient method to update certain parameters in an existing `Context` without needing to reinitialize it. Simply call `setBondParameters()` to modify this object’s parameters, then call `updateParametersInContext()` to copy them over to the `Context`.

        This method has several limitations. The only information it updates is the values of per-bond parameters. All other aspects of the `Force` (such as the energy function) are unaffected and can only be changed by reinitializing the `Context`. Neither the definitions of groups nor the set of groups involved in a bond can be changed, nor can new bonds be added.

    bool `usesPeriodicBoundaryConditions`() *const*¶
    :   Returns whether or not this force makes use of periodic boundary conditions.

        |  |  |
        | --- | --- |
        | Returns: | false |

High performance molecular simulation on GPUs


### Navigation

- Core Objects
- Forces
- Integrators
- Extras

©2015, Stanford University and the Authors.
|
Powered by Sphinx 1.3.1
& Alabaster 0.7.7
|
Page source
